# Examining Associations of HIV and Iron Status with Nutritional and Inflammatory Status, Anemia, and Dietary Intake in South African Schoolchildren

**DOI:** 10.3390/nu13030962

**Published:** 2021-03-16

**Authors:** Charlene Goosen, Jeannine Baumgartner, Nadja Mikulic, Shaun L. Barnabas, Mark F. Cotton, Michael B. Zimmermann, Renée Blaauw

**Affiliations:** 1 Division of Human Nutrition, Department of Global Health, Stellenbosch University, Cape Town 7505, South Africa; rb@sun.ac.za; 2 Laboratory of Human Nutrition, Institute of Food, Nutrition and Health, Department of Health Sciences and Technology, ETH Zurich, 8092 Zurich, Switzerland; jeannine.baumgartner@hest.ethz.ch (J.B.); nadja.mikulic@hest.ethz.ch (N.M.); michael.zimmermann@hest.ethz.ch (M.B.Z.); 3 Family Centre for Research with Ubuntu, Department of Pediatrics and Child Health, Stellenbosch University, Cape Town 7505, South Africa; barnabas@sun.ac.za (S.L.B.); mcot@sun.ac.za (M.F.C.)

**Keywords:** HIV, iron deficiency, anemia, nutritional status, stunting, dietary intake, children, South Africa

## Abstract

The etiology of multifactorial morbidities such as undernutrition and anemia in children living with the human immunodeficiency virus (HIV) (HIV+) on antiretroviral therapy (ART) is poorly understood. Our objective was to examine associations of HIV and iron status with nutritional and inflammatory status, anemia, and dietary intake in school-aged South African children. Using a two-way factorial case-control design, we compared four groups of 8 to 13-year-old South African schoolchildren: (1) HIV+ and low iron stores (inflammation-unadjusted serum ferritin ≤ 40 µg/L), *n* = 43; (2) HIV+ and iron sufficient non-anemic (inflammation-unadjusted serum ferritin > 40 µg/L, hemoglobin ≥ 115 g/L), *n* = 41; (3) children without HIV (HIV-ve) and low iron stores, *n* = 45; and (4) HIV-ve and iron sufficient non-anemic, *n* = 45. We assessed height, weight, plasma ferritin (PF), soluble transferrin receptor (sTfR), plasma retinol-binding protein, plasma zinc, C-reactive protein (CRP), α-1-acid glycoprotein (AGP), hemoglobin, mean corpuscular volume, and selected nutrient intakes. Both HIV and low iron stores were associated with lower height-for-age *Z*-scores (HAZ, *p* < 0.001 and *p* = 0.02, respectively), while both HIV and sufficient iron stores were associated with significantly higher CRP and AGP concentrations. HIV+ children with low iron stores had significantly lower HAZ, significantly higher sTfR concentrations, and significantly higher prevalence of subclinical inflammation (CRP 0.05 to 4.99 mg/L) (54%) than both HIV-ve groups. HIV was associated with 2.5-fold higher odds of iron deficient erythropoiesis (sTfR > 8.3 mg/L) (95% CI: 1.03–5.8, *p* = 0.04), 2.7-fold higher odds of subclinical inflammation (95% CI: 1.4–5.3, *p* = 0.004), and 12-fold higher odds of macrocytosis (95% CI: 6–27, *p* < 0.001). Compared to HIV-ve counterparts, HIV+ children reported significantly lower daily intake of animal protein, muscle protein, heme iron, calcium, riboflavin, and vitamin B_12_, and significantly higher proportions of HIV+ children did not meet vitamin A and fiber requirements. Compared to iron sufficient non-anemic counterparts, children with low iron stores reported significantly higher daily intake of plant protein, lower daily intake of vitamin A, and lower proportions of inadequate fiber intake. Along with best treatment practices for HIV, optimizing dietary intake in HIV+ children could improve nutritional status and anemia in this vulnerable population. This study was registered at clinicaltrials.gov as NCT03572010.

## 1. Introduction

Worldwide, an estimated 1.7 million children younger than 15 years old are living with the human immunodeficiency virus (HIV) (HIV+), with sub-Saharan Africa most affected [1]. Children with perinatally acquired HIV benefit from very early diagnosis and treatment [2,3], which significantly improves mortality and reduces morbidity [4,5]. Nevertheless, HIV+ children remain vulnerable to complications related to HIV or lifelong antiretroviral therapy (ART) [6]. Compared to individuals without HIV (HIV-ve) and despite viral suppression, HIV+ individuals have increased mortality risk that is associated with chronic inflammation [7].

Undernutrition persists among South African children [8,9,10,11,12], and disease, inadequate dietary intake, and poor nutrient bioavailability are possible causes [13]. Early in the HIV epidemic before ART, HIV contributed to severe undernutrition [14]. In developing countries, inadequate dietary intake stems from household food insecurity caused by complex social and economic challenges. Food insecurity affects diet quality, with higher intake of affordable plant staples and limited intake of nutrient-dense foods such as fruit, vegetables, and animal products. While such a diet may meet energy needs, it does not meet all nutrient requirements [15] and micronutrient deficiencies and anemia may develop [16].

Anemia affects several billion people globally, with the highest burden in developing countries and among children [17]. Iron deficiency is considered the most common cause of nutritional anemia [16,17]. Despite ART, anemia remains a common comorbidity in HIV+ individuals [18,19]. In addition to nutritional anemia, HIV+ individuals are at increased risk of anemia of chronic disease, a consequence of iron redistribution in the presence of systemic inflammation or chronic disease [20]. HIV also appears to directly induce dysregulation of erythropoiesis (red blood cell production) [19,21], and antiretrovirals such as zidovudine are associated with macrocytosis with or without concomitant anemia [21,22]. Anemic HIV+ individuals have an increased risk of all-cause mortality [23].

The etiology of undernutrition and anemia in children with perinatal HIV on ART is still poorly understood. We therefore examined associations of HIV and iron status with nutritional and inflammatory status, anemia, and dietary intake in school-aged South African children.

## 2. Materials and Methods

### 2.1. Study Design and Participants

This study followed a two-way factorial case-control design and was nested within a cohort of virally suppressed HIV+ and HIV-ve schoolchildren, purposefully selected to have low iron stores (serum ferritin ≤ 40 µg/L) or sufficient iron stores and no anemia (serum ferritin > 40 µg/L, hemoglobin (Hb) ≥ 115 g/L), for a series of iron studies at the Family Centre for Research with Ubuntu (FAMCRU) in Cape Town, South Africa. Children were enrolled using inflammation-unadjusted serum ferritin concentrations. Since low-grade inflammation was probable, especially among HIV+ children, a higher ferritin cut-off value for defining low iron stores was used at screening [24]. Children (*n* = 180) were enrolled into one of four distinct groups: (1) HIV+ and low iron stores, *n* = 45; (2) HIV+ and iron sufficient non-anemic, *n* = 45; (3) HIV-ve and low iron stores, *n* = 45; (4) HIV-ve and iron sufficient non-anemic, *n* = 45. Children from similar communities were recruited by community outreach teams, from previous research cohorts, or from the Tygerberg Hospital infectious diseases outpatient unit. Children were screened and enrolled between September 2018 and August 2019. Inclusion criteria for screening were: (1) age 8–13 years; (2) no current self-reported acute illness; (3) no iron-containing supplementation use in the three months preceding the visit; and (4) HIV plasma viral load < 50 copies/mL in HIV+ children (from routine care electronic health records). Screening procedures included anthropometrics and a venipuncture, and exclusion criteria for enrollment were: (1) severe underweight (body-mass-index-for-age *Z*-score (BAZ) < −3) or obesity (BAZ > 2) [25], and (2) severe anemia (Hb < 80 g/L) [26]. The inclusion criterion for the low iron store groups was inflammation-unadjusted serum ferritin ≤ 40 µg/L; and inclusion criteria for the iron sufficient non-anemic groups were inflammation-unadjusted serum ferritin > 40 µg/L and Hb ≥ 115 g/L. In the HIV-ve children, the absence of HIV was confirmed with a rapid HIV assay (First Response HIV Card 1–2.0, Premier Medical Corporation Pvt Ltd., Sarigam, India).

For this present study we used baseline data of all enrolled participants. At baseline measured viral loads were ≥50 copies/mL in six children who were then excluded from the analyses. Thus, the final groups for this study were: (1) HIV+ and low iron stores, *n* = 43; (2) HIV+ and iron sufficient non-anemic, *n* = 41; (3) HIV-ve and low iron stores, *n* = 45; (4) HIV-ve and iron sufficient non-anemic, *n* = 45. Based on the two-way factorial design, assuming 80% power and a type I error rate of 5%, the final sample size allowed us to detect a medium to large effect size of 0.3 between groups.

### 2.2. Socio-Demographic and Anthropometric Indicators

We obtained socio-demographic information from the children and their caregivers using a structured questionnaire. Anthropometric measurements were performed using standardized techniques [27]. Weight and height were measured using a Micro 1023 electronic platform scale and stadiometer (Scalerite, Johannesburg, South Africa). Children were measured barefoot in a single layer of clothing. When applicable, hair was undone, and hair accessories removed. Weight was measured to the nearest 0.1 kg and height to the last completed 0.1 cm.

### 2.3. Biochemical Measurements

In all children, the study nurses drew a venous blood sample into EDTA and serum-separating tubes (SST) BD vacutainers (Becton, Dickinson and Company, Franklin Lakes, NJ, USA), as well as into trace metal free lithium-heparin coated vacutainers (Sarstedt, Nümbrecht, Germany). The time of blood draw and the time of the last meal were recorded. On the day of blood draw, Hb concentrations and red blood cell morphology were measured in whole blood using a Siemens Advia 2120i Hematology System, (Siemens, Munich, Germany) and serum ferritin was measured using the Roche COBAS Elecsys Ferritin assay (Hoffmann-La Roche, Basel, Switzerland). Plasma was separated from the remaining blood, aliquoted for micronutrient, inflammatory marker, and HIV viral load measurements, and then frozen at −70 °C. HIV viral loads were measured using the Roche COBAS AmpliPrep/TaqMan HIV-1 Test, v2 (Hoffmann-La Roche, Basel, Switzerland). We measured iron status (plasma ferritin (PF) and soluble transferrin receptor (sTfR)), vitamin A status (plasma retinol-binding protein (RBP)), and inflammation (C-reactive protein (CRP) and α-1-acid glycoprotein (AGP)) using a multiplex immunoassay previously described [28]. To minimize environmental contamination, aliquots for zinc analysis were stored in Eppendorf tubes pre-washed in 10% nitric acid (65%, Merck KGaA, Darmstadt, Germany), diluted with nanopure water for at least 6 h, and afterwards washed in nanopure water for at least 4 h. We quantitatively determined plasma zinc (PZn) content with a flame atomic absorption spectrometer (AA240FS; Varian, Inc, Palo Alto, CA) with a commercial aqueous Titrisol standard (Merck KGaA, Darmstadt, Germany) for external calibration and wheat flour (SRM 1567a, US National Institute of Standards and Technology) as control material, as previously reported [29].

### 2.4. Dietary Intake Assessment

We collected dietary intake information using an abbreviated quantified food frequency questionnaire (aQFFQ) and portion size estimation kit developed for the specific study population [30]. The aQFFQ food list included fortified wheat and maize products, fiber-rich grains and cereals, nuts, fruit, vegetables, legumes, dairy, eggs, and animal flesh products that reflected the study population’s habitual intake. The food list excluded basic non-fortified low-fiber staples (such as pasta and white rice), sugar and sugary foods, fats, oils, and salt. The aQFFQ could therefore estimate intake of protein, fiber, and selected micronutrients (iron, zinc, calcium, vitamin A, thiamine, riboflavin, niacin, vitamin B_6_, folate, vitamin B_12_, and vitamin C). Household measures were quantified according to the Food Quantities Tables for South Africa [31] and coded using the Food Composition Tables for South Africa [32]. Consumption for the past month (28 days) was reported. Frequency reporting was done as number of times per day, per week, or per month. Portion size estimation was facilitated with food portion photographs, various crockery and household utensil sizes, and pre-weighed food examples specific to the aQFFQ. The aQFFQ was interview-administered, once-off, by a single trained nutrition professional with the child-caregiver pair. Interviews were conducted in the child and caregiver’s preferred language and where necessary, a translator from the research site assisted.

### 2.5. Data Management and Definitions

With the exception of dietary intake data, all study data were collected and managed using REDCap electronic data capture tools hosted at the ETH Zurich [33,34]. Height-for-age *Z*-scores (HAZ) and BAZ were calculated using the WHO AnthroPlus Software Version 1.0.4. Stunting was defined as HAZ < −2. Underweight was defined as BAZ ≥ −3 and < −2, and overweight as BAZ > 1 and ≤ 2 [25].

We adjusted PF, RBP and PZn values for inflammation using the regression correction approach proposed by the Biomarkers Reflecting Inflammation and Nutritional Determinants of Anemia (BRINDA) group. This approach accounts for the magnitude of inflammation measured by CRP and AGP, and therefore allows for more precise adjustments of acute phase proteins [35,36]. Iron deficiency (ID) was defined as adjusted PF < 15 µg/L [24]. Iron deficient erythropoiesis was defined as sTfR > 8.3 mg/L [28]. Vitamin A deficiency was defined as adjusted RBP < 0.70 µmol/L and mild vitamin A deficiency as adjusted RBP concentrations of 0.7 to 1.05 µmol/L [37]. Zinc deficiency was defined according to age, sex, time of day, and time of last meal as proposed by the International Zinc Nutrition Consultative Group (IZINCG) [38]. Children were not expected to arrive fasting, but within our resource-limited setting some did. Zinc deficiency was defined as adjusted PZn below the following cut-off values: For non-fasting morning blood draws, 65 µg/dL for males and females younger than 10 years, 66 µg/dL for females 10 years and older, and 70 µg/dL for males 10 years and older. For fasting morning blood draws, 70 µg/dL for females 10 years and older, and 74 µg/dL for males 10 years and older [38]. Anemia was defined as Hb < 115 g/L for children 8 to 11 years old, and Hb < 120 g/L for children 12 to 13 years old. Moderate anemia was defined as Hb concentrations of 80 to 109 g/L, and mild anemia was defined as Hb concentrations of 110 to 114 g/L for children 8 to 11 years old, and 110 to 119 g/L for children 12 to 13 years old [26]. Iron deficiency anemia was defined as adjusted PF < 15 µg/L and/or sTfR > 8.3 mg/L, and anemia. Microcytosis, normocytosis, and macrocytosis were defined as a mean corpuscular volume (MCV) of <77.1 fL, 77.1 to 91.5 fL, and >91.5 fL, respectively, for children aged 8 to 12 years old, as a MCV of <83.1 fL, 83.1 to 101.6 fL, and >101.6 fL, respectively, for 13-year-old males, and as a MCV of <78.9 fL, 78.9 to 98.5 fL, and >98.5 fL, respectively, for 13-year-old females (cut-off values used by the National Health Laboratory Service in South Africa). Concomitant with anemia, microcytic, normocytic, and macrocytic anemia were defined, respectively. The presence of inflammation was classified as CRP ≥ 5 mg/L or AGP > 1 g/L. CRP concentrations of 0.05 to 4.99 mg/L were classified as subclinical inflammation, reflecting detectable CRP concentrations below the clinically used threshold for acute infections.

From the reported dietary intake portions and frequencies, daily food intake quantities were calculated and captured using Microsoft Excel (Microsoft, Redmond, WA, USA). Dietary intake data were converted to nutrients using the South African Food Data System (SAFOODS) Food Composition Database Version 2019 [39]. The data system includes the fortified nutrients of the mandated South African food fortification program. We estimated the prevalence of inadequate nutrient intake using the estimated average requirements (EAR) cut-point method, whereby the proportion of children with nutrient intakes below the EAR is calculated [40,41]. The EAR is the daily intake level per nutrient estimated to meet the needs of half the individuals in age and sex-specific population groups. In the absence of established EAR values for fiber, adequate intake (AI) values were used [40].

### 2.6. Statistical Analyses

Statistical analyses were performed using IBM SPSS Statistics version 27 (SPSS Inc., Chicago, IL, USA). Participant characteristics were summarized with descriptive statistics. Categorical variables are reported as frequencies and percentages. The distribution of continuous variables was investigated using Q–Q plots and the Shapiro–Wilk test. Homogeneity of variance was tested with the Levene’s test. Normally distributed continuous variables are reported as means and standard deviations (SD), and non-normally distributed continuous variables are reported as medians and inter-quartile ranges (IQR). Non-normally distributed outcome variables were log-transformed before analyses. We assessed associations of the factors HIV and iron status with socio-demographic outcome variables, using two-way factorial analysis of variance (ANOVA) for continuous outcome variables, and two-way binary logistic regression for categorical outcome variables. We assessed associations of HIV and iron status with outcome variables (anthropometrics, micronutrients, inflammation, anemia, and dietary intake) using two-way factorial analysis of covariance (ANCOVA) for continuous variables, and two-way binary logistic regression for categorical variables, adjusting for age and sex. If the logistic regression model showed no significant interaction effect, it was repeated without the interaction factor. If the two-way ANCOVA or logistic regression model showed a significant HIV, iron status, or interaction effect, we performed a between-group comparison using one-way ANOVA and Chi-square test, respectively, with Bonferroni adjustment for multiple comparisons. We report adjusted odds ratios (OR) and 95% confidence intervals (CI) for the regression parameters. Statistical significance was set at *p* < 0.05.

## 3. Results

### 3.1. Participant Characteristics and Socio-Demographic Indicators

Of 293 children screened, 180 children were enrolled. Data from 174 children, 84 HIV+ and 90 HIV-ve, were included for analysis (six HIV+ children were excluded because of detectable viral loads). Table 1 presents the participant and household socio-demographic characteristics by group. The HIV+ children were older (*p* = 0.04) than the HIV-ve children and zidovudine-containing ART regimens were more prevalent among iron sufficient non-anemic HIV+ children (*p* = 0.01). In Appendix A, Table A1 lists all ART regimens. All children attended schools that offered the National School Nutrition Program. Significantly more HIV+ children (*p* = 0.006) and children with low iron stores (*p* = 0.003) accessed the National School Nutrition Program than their HIV-ve and iron sufficient non-anemic counterparts. It is often difficult to obtain information on household income and in our study, 25% of caregivers chose not to disclose their monthly incomes. Differences in proxy indicators of socio-economic status and financial security indicated that HIV+ children were from smaller households (*p* = 0.02) with borderline lower permanently employed breadwinners (*p* = 0.05), and more children with low iron stores were in the care of single primary caregivers (*p* = 0.02) and resided in households with fewer temporary employed breadwinners (*p* = 0.04).

### 3.2. Anthropometric Status

Both HIV and low iron stores were associated with lower HAZ (*p* < 0.001 and *p* = 0.02, respectively) (Table 2). Subsequently, HAZ differed significantly between HIV+ children with low iron stores and both HIV-ve groups. The highest proportion of stunting was among the HIV+ children with low iron stores (30%), albeit no significant main effect for HIV and iron status, and no significant HIV × iron status interaction (*p* = 0.36) (data not shown). BAZ was borderline lower in the HIV+ children (*p* = 0.06).

### 3.3. Micronutrient Deficiencies and Inflammation

One-third (34%) of the HIV+ and HIV-ve children enrolled into the low iron store groups (inflammation-unadjusted serum ferritin ≤40 µg/L) were iron deficient (adjusted PF < 15 µg/L) (Table 3). HIV was associated with higher sTfR concentrations (*p* = 0.003) and higher proportions of iron deficient erythropoiesis (OR: 2.5; 95% CI: 1.03–5.8; *p* = 0.04). HIV+ children with low iron stores had significantly higher sTfR concentrations than both HIV-ve groups. We found no difference in vitamin A or zinc status by HIV or iron status. In total, 10% of all children were vitamin A deficient (adjusted RBP < 0.7 µmol/L) and 50% presented with mild vitamin A deficiency (adjusted RBP 0.7 to 1.05 µmol/L). Zinc deficiency was present in 45% of all children. Both HIV and sufficient iron stores were associated with significantly higher CRP and AGP concentrations, resulting in significant differences between iron sufficient non-anemic HIV+ children and HIV-ve children with low iron stores. Subclinical inflammation (CRP 0.05 to 4.99 mg/L) was more prevalent in HIV+ than HIV-ve children (OR: 2.7; 95% CI: 1.4–5.3; *p* = 0.004), and significantly higher in HIV+ iron deficient children (54%) than in both HIV-ve groups. CRP concentrations ≥ 5 mg/L were more prevalent in iron sufficient non-anemic children (OR: 12; 95% CI: 1.4–95; *p* = 0.02) than in children with low iron stores.

### 3.4. Anemia

Among those in the low iron store groups, 35% of HIV+ children and 18% of HIV-ve children were anemic, with mostly mild anemia (Table 4). Macrocytosis occurred in 67% of the HIV+ children. HIV was associated with higher MCV (*p* < 0.001), resulting in significantly higher MCV in both HIV+ groups than their HIV-ve counterparts. However, HIV+ children with low iron stores had significantly lower MCV than HIV+ iron sufficient non-anemic children, which resulted in a significant HIV × iron status interaction (*p* = 0.01). Consequently, HIV was associated with higher proportions of macrocytosis (OR: 12; 95% CI: 6–27; *p* < 0.001) and macrocytic anemia (OR: 15; 95% CI: 1.7–121; *p* = 0.01). In this sample, zidovudine-containing ART was significantly associated with macrocytosis (*p* = 0.008) (data not shown).

### 3.5. Selected Nutrient Intake

Nutrient inadequacy, measured using the EAR cut-point method, was virtually absent for total protein and the micronutrients iron, zinc, thiamin, niacin, vitamin B_6_ and vitamin B_12_ (Table 5). The greatest proportions of nutrient inadequacy were for calcium, vitamin A, vitamin C, and fiber. HIV+ children had lower daily intake of animal protein (*p* < 0.001), muscle protein (*p* < 0.001), heme iron (*p* < 0.001), calcium (*p* < 0.001), riboflavin (*p* < 0.001), and vitamin B_12_ (*p* < 0.001), and higher proportions of inadequate vitamin A (*p* = 0.049) and fiber intake (*p* = 0.04). Children with low iron stores had higher daily intake of plant protein (*p* = 0.048), lower daily intake of vitamin A (*p* = 0.02), and lower proportions of inadequate fiber intake (*p* = 0.03). Daily intake of iron and zinc from fortified foods was higher in HIV+ children (*p* = 0.002 and *p* < 0.001, respectively) and lower (or borderline lower) in iron sufficient non-anemic children (*p* = 0.03 and *p* = 0.06, respectively) if HIV-ve. This resulted in a significant HIV × iron status interaction (*p* = 0.02 and *p* = 0.007, respectively), and HIV-ve iron sufficient non-anemic children having significantly lower intake of iron and zinc from fortified foods than the other three groups. HIV+ children had lower vitamin C intake (*p* < 0.001) but when comparing groups, this effect was only observed in the iron sufficient non-anemic children, resulting in a significant HIV × iron status interaction (*p* = 0.004). The proportion of children not meeting the EAR for vitamin C was higher among HIV+ children if iron sufficient non-anemic, and lower in HIV-ve children if iron sufficient non-anemic, resulting in a significant HIV × iron status interaction (*p* < 0.001).

The most frequently eaten food items from the aQFFQ (fortified staples, nutrient-dense foods, and fiber-rich foods) are shown in Appendix B, Table A2. Foods are ranked by the proportion of children who consumed the foodstuff, as well as by mean daily intake (excluding items consumed by <5% of the children). Nearly all children ate bread and maize meal, two of the food vehicles used for fortification in South Africa. The animal products consumed by most children included chicken, polony (cold meat sausage made from a combination of beef and pork), and fish as muscle protein sources, and milk and eggs as further animal protein sources.

## 4. Discussion

In this cohort of 8 to 13-year-old virally suppressed HIV+ and HIV-ve South African children selected to have low iron stores or sufficient iron stores and no anemia, both HIV and low iron stores were associated with lower stature, while both HIV and sufficient iron stores were associated with significantly higher inflammation. HIV+ children with low iron stores had significantly lower HAZ, significantly higher sTfR concentrations, and significantly higher prevalence of subclinical inflammation than both HIV-ve groups. HIV was associated with significantly higher odds of iron deficient erythropoiesis, subclinical inflammation, and macrocytosis. Compared to HIV-ve counterparts, HIV+ children reported significantly lower daily intake of animal protein, muscle protein, heme iron, calcium, riboflavin, and vitamin B_12_, and significantly higher proportions of HIV+ children did not meet vitamin A and fiber requirements. Compared to iron sufficient non-anemic counterparts, children with low iron stores reported significantly higher daily intake of plant protein, lower daily intake of vitamin A, and lower proportions of inadequate fiber intake.

In a previous prospective cohort study, Shet and colleagues followed 240 perinatal HIV+ children (2 to 12 years old) of whom 43% were on ART. They reported iron deficiency, vitamin A deficiency, and chronic inflammation as the major determinants of anemia in their cohort and found inadequate dietary iron intake among 78% of children [42]. Shorter stature without underweight (BAZ < −2) in HIV+ infants and children has been reported before [43,44]. Cohort studies have reported significantly poorer growth during the first few months of life in HIV+ compared to HIV-ve infants when ART is initiated late [44,45], as in the present study. The 8 to 13-year-old children in our study were born when the HIV program was rapidly evolving, when ART initiation in pregnant women was still driven by clinical and CD4-based criteria [46], and when many HIV+ women formula fed despite the lack of resources [47]. These children also started ART far later than below three months of age and more recently, close to birth. This potentially reflects higher vulnerability and exposure to stunting risk factors and may explain, at least in part, the significant association between HIV and height deficits in this age group. Significant associations between children with low iron stores and HAZ potentially highlight longstanding exposure to inadequate dietary intake and undernutrition.

HIV was associated with significantly higher prevalence of iron deficient erythropoiesis. HIV was also associated with significantly lower dietary intake of hemopoietic nutrients (animal protein, muscle protein, heme iron, riboflavin, and vitamin B_12_) and significantly higher prevalence of subclinical inflammation, both potential causes of iron deficient erythropoiesis [16,48,49,50]. Zidovudine-containing ART regimens were significantly associated with macrocytosis, a common finding among HIV+ individuals receiving ART and regarded benign [22]. The HIV × iron status interaction highlights the opposing effect of HIV and iron deficiency on MCV: HIV was associated with significantly higher MCV (both groups vs. HIV-ve groups), but when HIV+ children also had low iron stores, their MCV was significantly lower than in HIV+ iron sufficient non-anemic children. Since ID is typically associated with microcytosis, higher MCV in HIV+ children could mask iron deficiency anemia if evaluated using only a full blood count. Other potential causes of macrocytic anemia include folate and vitamin B_12_ deficiencies [16]. While habitual dietary intake of vitamin B_12_ was significantly lower among the HIV+ children, we did not measure vitamin B_12_ status and therefore cannot determine its contribution. Vitamin B_12_ deficiency does not appear to be common in children living with HIV. In a previous study among children with perinatal HIV and high prevalence of anemia, ID, and vitamin A deficiency, vitamin B_12_ deficiency was only present in 8% of children [42]. Considering other causes of anemia, combined vitamin A and mild vitamin A deficiency (adjusted RBP ≤ 1.05 µmol/L) was prevalent in 60% of children in the present study and could potentially have contributed to the pathophysiology of anemia.

There were significant differences in habitual dietary intake that highlight increased vulnerability to undernutrition among the HIV+ children. Compared to their HIV-ve counterparts, they consumed less nutrient-dense foods of animal origin, reflected by significantly lower daily intake of calcium (dairy), muscle protein, heme iron, riboflavin, and vitamin B_12_ (animal flesh), and higher proportions of inadequate vitamin A intake (especially abundant in organ meat). A similar dietary intake vulnerability can be deduced for children with low iron stores, who had higher intake of plant protein corresponding to lower proportions of inadequate fiber intake, and lower intake of vitamin A. The intake of plant and fortified foods enabled the children to meet iron and zinc requirements, but these less bioavailable forms of iron and zinc did not translate to eradicating deficiency states. More HIV+ children and children with low iron stores accessed the National School Nutrition Program, which may reflect higher dependence on this program aimed at alleviating hunger among schoolchildren [51]. While the National School Nutrition Program contributes to nutrient intake, animal foods are not supplied in abundance and the intake of these less affordable products rely on household food security. The sociodemographic household characteristics did not reflect clear disparities by HIV or iron status. More single primary caregivers among the children with low iron stores could suggest less financial stability in the absence of a partner who contributes to the household income. Significantly less temporary employment and borderline lower permanent employment among household breadwinners of children with low iron stores and HIV+ children, respectively, also suggest potentially lower household income which could adversely affect household food security and dietary diversity. In Johannesburg, South Africa, poor dietary diversity was recently described among 1 to 10-year-old HIV+ children [52], while a previous study comparing 5 to 9-year-old HIV+ and HIV-ve children revealed similar borderline-low dietary diversity scores in both groups [53]. Of concern is the lower intake of muscle protein/heme iron by the HIV+ children. Muscle protein/heme iron is an important dietary determinant of iron status, and the only dietary component that is consistently positively associated with iron stores [54]. A prospective study among virally suppressed HIV+ South African children investigated differences in dietary intake by iron status. They concluded that dietary iron intake did not protect against iron deficiency, while daily intake of >20 g animal protein had a protective effect [55]. Muscle protein not only provides highly bioavailable heme iron, but also enhances the absorption of non-heme iron [54]. In resource-limited settings, intervention approaches often focus on the enhancing effects of dietary components such as ascorbic acid or inhibitory effects of phytates, polyphenols and calcium. Their potential effects on iron absorption are dose-dependent [50], and their interplay with dietary iron stretch to whole diets and dietary patterns [54]. While using a range of strategies to improve iron status remains important, investing in small quantities of heme-iron appear vital, and will simultaneously increase the intake of nutrients such as zinc, vitamin A, riboflavin, and vitamin B_12_. Household strategies include purchasing more affordable muscle proteins, and small-livestock production and aquaculture where feasible [56]. Recently, the need for re-engineering food systems and cost drivers were highlighted, to bring down the cost of healthy nutrient-dense food and increase the purchasing power of poorer communities [15].

A strength of our study is the large array of biomarkers investigated to examine the associations of HIV and iron status with nutritional status, inflammation, anemia, and dietary intake in virally suppressed HIV+ versus HIV-ve children. Furthermore, obtaining dietary data, a component often excluded since it is resource intense, provided insight into dietary vulnerabilities among HIV+ children where comorbidities such as anemia may be attributed to chronic disease or inflammation. However, the findings should be interpreted within the study’s limitations. The observational design of the study does not allow conclusions on causality of the associations observed. The sample size allowed us to detect associations of medium to large effects size, and findings should be confirmed in larger samples. Based on the enrollment criteria of the sample, our interpretations regarding anthropometric status are limited by the exclusion of severely underweight and obese children. Furthermore, since only a third of children in the low iron store groups were truly iron deficient, there may be more between-group difference by iron status than reported. Nevertheless, vulnerability by iron status and the need for improving dietary intake was still evident. Our use of an aQFFQ has some limitations. Since we only obtained information on selected food intake, we cannot assess the adequacy of energy intake. Reporting relies on participant recall, and the risk of overestimating dietary intake is higher with a food frequency questionnaire compared to other methods [57]. A strength of using the aQFFQ compared to other methods is that it better reflects habitual dietary intake, especially for nutrients such as vitamin A present in high concentrations in only a few foods [58].

## 5. Conclusions

While ART offers HIV+ children a lifeline to a healthy life, they remain vulnerable to the effects of chronic disease and lifelong drug treatment, such as subclinical inflammation and macrocytosis, and appear more vulnerable to dietary determinants of undernutrition and anemia, such as poorer dietary intake. Further to ART and best treatment practices, optimal nutrition in HIV+ individuals plays an important role in reducing comorbidity risk and improving health outcomes [59]. Dietary intake is a modifiable factor, and food-based strategies remain the most sustainable approaches to preventing micronutrient deficiencies [60]. The intake of muscle protein/heme iron remains vital for preventing ID in schoolchildren. It gains even more importance in chronic diseases where the risk of ID may be higher, and where potential risks associated with therapeutic oral iron supplementation for treating deficiency [23] warrant prioritization of prevention strategies.

## Figures and Tables

**Table 1:** Participant and household socio-demographic characteristics in the four groups of South African children purposefully selected according to human immunodeficiency virus (HIV) and iron status.

	HIV+ and Low Iron Stores ^1^	HIV+ and Iron Sufficient Non-Anemic	HIV-ve and Low Iron Stores	HIV-ve and Iron Sufficient Non-Anemic	*p*-Value ^2^	
	*n* = 43	*n* = 41	*n* = 45	*n* = 45	HIV	Iron Status
Male/female, *n* (%)	20 (47)/23 (54)	27 (66)/14 (34)	22 (49)/23 (51)	24 (53)/21 (47)	0.51	0.12
Age,^3^ y	12 (10–12)	11 (11–12)	11 (10–12)	10 (9–12)	0.04	0.85
Age at antiretroviral therapy start,^4^ y	1 (0–2.5)	1 (0–1)	-	-	-	0.75
Zidovudine-containing antiretroviral regimen, *n* (%)	5 (12)	15 (37)	-	-	-	0.01
Menstruating females, *n* (%)	7 (30)	0	2 (9)	5 (24)	0.77	0.56
Access school nutrition program, *n* (%)	40 (93)	34 (83)	38 (84)	26 (58)	0.006	0.003
Formal/informal housing,^5^ *n* (%)	26 (61)/17 (40)	23 (56)/18 (44)	31 (69)/14 (31)	31 (69)/14 (31)	0.15	0.77
Number of household members	5 (4–6)	5 (4–6)	5 (4–7)	6 (5–7)	0.02	0.27
Primary caregiver single/in partnership, *n* (%)	24 (56)/19 (44)	16 (39)/25 (61)	20 (44)/25 (56)	12 (27)/33 (73)	0.11	0.02
Breadwinner permanent employment, *n* (%)	13 (30)	9 (22)	17 (38)	19 (42)	0.05	0.81
Breadwinner temporary employment, *n* (%)	10 (23)	15 (37)	6 (13)	12 (27)	0.13	0.04
Breadwinner unemployed, *n* (%)	20 (47)	17 (42)	22 (49)	14 (31)	0.60	0.12
Household receives government grant, *n* (%)	39 (91)	39 (95)	41 (91)	38 (84)	0.27	0.77

^1^ Study group enrollment criteria used: Low iron stores if inflammation-unadjusted serum ferritin ≤ 40 µg/L; and iron sufficient non-anemic if inflammation-unadjusted serum ferritin > 40 µg/L and hemoglobin concentration ≥ 115 g/L. ^2^ Associations of the factors HIV and iron status with the participant and household characteristics were assessed using two-way analysis of variance (ANOVA) for continuous variables and two-way logistic regression analysis for categorical variables. Interaction effect *p*-values are omitted since none were significant. ^3^ Median (inter-quartile range) (all such values). ^4^ HIV and low iron stores *n* = 39, and HIV and iron sufficient non-anemic *n* = 39, as start date unknown for three children. ^5^ Formal housing represents a brick house, while informal housing represents a Wendy house or dwelling built with scrap building material and typically not equipped with water and/or electricity.

**Table 2:** Anthropometric status in the four groups of South African children purposefully selected according to HIV and iron status.

	HIV+ and Low Iron Stores ^1^	HIV+ and Iron Sufficient Non-Anemic	HIV-ve and Low Iron Stores	HIV-ve and Iron Sufficient Non-Anemic	*p*-Value ^2^	
	*n* = 43	*n* = 41	*n* = 45	*n* = 45	HIV	Iron Status
Height-for-age *Z*-score ^3^	−1.4 ± 1 ^a^	−1.1 ± 0.9 ^ab^	−0.7 ± 1 ^bc^	−0.4 ± 0.9 ^c^	<0.001	0.02
Stunted,^4^ *n* (%)	13 (30)	6 (15)	6 (13)	4 (9)	0.12	0.07
Body-mass-index-for-age *Z*-score	−0.4 ± 1	−0.5 ± 0.9	−0.3 ± 1.1	−0.1 ± 1	0.06	0.38
Underweight,^5^ *n* (%)	1 (2)	1 (2)	4 (9)	0	0.44	0.11
Overweight,^5^ *n* (%)	4 (9)	2 (5)	6 (13)	6 (13)	0.13	0.89

^1^ Study group enrollment criteria used: Low iron stores if inflammation-unadjusted serum ferritin ≤ 40 µg/L; and iron sufficient non-anemic if inflammation-unadjusted serum ferritin > 40 µg/L and hemoglobin concentration ≥115 g/L. ^2^ Associations of the factors HIV and iron status with the anthropometric indices were assessed using two-way analysis of covariance (ANCOVA) for continuous variables and two-way logistic regression analysis for categorical variables, adjusting for age and sex. Between-group differences were analyzed using one-way ANOVA with Bonferroni adjustment for multiple comparisons. Means in a row without a common letter (a, b, c) differ significantly, *p* < 0.05. Interaction effect *p*-values are omitted since none were significant. ^3^ Mean (standard deviation) (all such values). ^4^ Height-for-age *Z*-score < −2 [25]. ^5^ Underweight: body-mass-index-for-age *Z*-score (BAZ) ≥ −3 and <−2; Overweight: BAZ > 1 and ≤2 [25].

**Table 3:** Micronutrient status and inflammation in the four groups of South African children purposefully selected according to HIV and iron status.

	HIV+ and Low Iron Stores ^1^	HIV+ and Iron Sufficient Non-Anemic	HIV-ve and Low Iron Stores	HIV-ve and Iron Sufficient Non-Anemic	*p*-Value ^2^	
	*n* = 43	*n* = 41	*n* = 45	*n* = 45	HIV	Iron Status
Plasma ferritin (unadjusted),^3^ µg/L	18 (14–27) ^b^	45 (36–72) ^a^	20 (15–26) ^b^	41 (30–60) ^a^	0.28	<0.001
Plasma ferritin (adjusted),^4^ µg/L	17 (13–25) ^b^	44 (35–70) ^a^	20 (14–26) ^b^	40 (29–57) ^a^	0.50	<0.001
Iron deficiency,^5^ *n* (%)	16 (37) ^a^	0 ^b^	14 (31) ^a^	1 (2) ^b^	0.91	<0.001
Plasma soluble transferrin receptor, mg/L	7.0 (5.6–8.2) ^a^	6.8 (5.7–8.3) ^ab^	6.4 (5.4–7.4) ^b^	6.0 (5.0–7.0) ^b^	0.003	0.07
Iron deficient erythropoiesis,^6^ *n* (%)	9 (21)	10 (24)	5 (11)	4 (9)	0.04	0.99
Plasma retinol binding protein (unadjusted), µmol/L	0.95 (0.8–1.1)	0.97 (0.8–1.1)	0.96 (0.8–1.2)	0.87 (0.8–1.1)	0.80	0.81
Plasma retinol binding protein (adjusted),^7^ µmol/L	0.99 (0.8–1.1)	1.03 (0.9–1.1)	0.96 (0.8–1.2)	0.89 (0.8–1.2)	0.40	0.62
Vitamin A deficiency,^8^ *n* (%)	6 (14)	3 (7)	3 (7)	5 (11)	0.68	0.75
Vitamin A mild deficiency,^8^ *n* (%)	19 (44)	20 (49)	24 (53)	24 (53)	0.50	0.94
Plasma zinc (unadjusted), µg/dL	72 (65–80)	68 (60–76)	69 (63–76)	72 (63–76)	0.53	0.44
Plasma zinc (adjusted),^9^ µg/dL	72 (65–81)	70 (61–76)	70 (63–75)	72 (65–76)	0.41	0.43
Zinc deficiency,^10^ *n* (%)	16 (37)	21 (51)	22 (49)	19 (42)	0.74	0.62
Plasma C-reactive protein, mg/L	0.1 (0.02–0.9) ^ab^	0.1 (0.02–1.5) ^b^	0.03 (0.01–0.05) ^a^	0.04 (0.02–0.3) ^ab^	0.003	0.03
Plasma C-reactive protein 0.05–4.99 mg/L,^11^ *n* (%)	23 (54) ^a^	15 (37) ^ab^	11 (24) ^b^	11 (24)^b^	0.004	0.17
Plasma C-reactive protein ≥5 mg/L, *n* (%)	1 (2)	6 (15)	0	3 (7)	0.11	0.02
Plasma α-1-acid glycoprotein, g/L	0.5 (0.5–0.8) ^ab^	0.7 (0.5–0.8) ^a^	0.5 (0.4–0.6) ^b^	0.6 (0.4–0.7) ^ab^	0.03	0.04
Plasma α-1-acid glycoprotein >1 g/L, *n* (%)	5 (12)	5 (12)	0	5 (11)	0.10	0.17

^1^ Study group enrollment criteria used: Low iron stores if inflammation-unadjusted serum ferritin ≤ 40 µg/L; and iron sufficient non-anemic if inflammation-unadjusted serum ferritin >40 µg/L and hemoglobin concentration ≥ 115 g/L. ^2^ Associations of the factors HIV and iron status with the micronutrient and inflammation indices was assessed using two-way analysis of variance (ANCOVA) for continuous variables and two-way logistic regression analysis for categorical variables, adjusting for age and sex. Between-group differences were analyzed using one-way ANOVA or Chi-square tests with Bonferroni adjustment for multiple comparisons. Non-normally distributed variables were log-transformed prior to analysis. Medians in a row without a common letter (a, b) differ significantly, *p* < 0.05. Interaction effect *p*-values are omitted since none were significant. ^3^ Median (inter-quartile range) (all such values). ^4^ Plasma ferritin adjusted for inflammation [35]. ^5^ Adjusted plasma ferritin < 15 µg/L [24]. ^6^ Plasma soluble transferrin receptor > 8.3 mg/L [28]. ^7^ Plasma retinol binding protein adjusted for inflammation [35]. ^8^ Vitamin A deficiency: adjusted plasma retinol-binding protein < 0.7 µmol/L; Mild vitamin A deficiency: adjusted plasma retinol-binding protein of 0.7 to 1.05 µmol/L [37]. ^9^ Plasma zinc adjusted for inflammation [36]. ^10^ Adjusted plasma zinc below the following cut-off values: For non-fasting morning blood draws, 65 µg/dL for males and females younger than 10 years, 66 µg/dL for females 10 years and older, and 70 µg/dL for males 10 years and older. For fasting morning blood draws, 70 µg/dL for females 10 years and older, and 74 µg/dL for males 10 years and older [38]. ^11^ Plasma C-reactive protein: limit of detection = 0.05 mg/L.

**Table 4:** Hematological indices in the four groups of South African children purposefully selected according to HIV and iron status.

	HIV+ and Low Iron Stores ^1^	HIV+ and Iron Sufficient Non-Anemic	HIV-ve and Low Iron Stores	HIV-ve and Iron Sufficient Non-Anemic	*p*-Value ^2^	
	*n* = 43	*n* = 41	*n* = 45	*n* = 45	HIV	Iron Status
Hemoglobin,^3^ g/L	120 (10) ^b^	126 (7) ^a^	125 (12) ^ab^	128 (6) ^a^	0.02	0.001
Total anemia,^4^ *n* (%)	15 (35) ^a^	1 (2) ^b^	8 (18) ^ab^	1 (2) ^b^	0.11	<0.001
Moderate anemia,^4^ *n* (%)	4 (9)	0	3 (7)	0	0.64	1.00
Mild anemia,^4^ *n* (%)	11 (26) ^a^	1 (2) ^b^	5 (11) ^ab^	1 (2) ^b^	0.14	0.003
Iron deficiency anemia,^5^ *n* (%)	10 (23)	0	5 (11)	0	0.17	1.00
Mean corpuscular volume,^6^ fL	94 (10) ^b^	100 (10) ^a^	87 (4) ^c^	87 (6) ^c^	<0.001	0.02
Microcytosis,^7^ *n* (%)	3 (7)	0	0	2 (4)	0.48	0.73
Normocytosis,^7^ *n* (%)	14 (33) ^b^	11 (27) ^b^	39 (87) ^a^	35 (78) ^a^	<0.001	0.17
Macrocytosis,^7^ *n* (%)	26 (61) ^a^	30 (73) ^a^	6 (13) ^b^	8 (18) ^b^	<0.001	0.13
Microcytic anemia,^8^ *n* (%)	2 (5)	0	0	0	1.00	1.00
Normocytic anemia,^8^ *n* (%)	3 (7)	0	7 (16)	1 (2)	0.13	0.02
Macrocytic anemia,^8^ *n* (%)	10 (23) ^a^	1 (2) ^b^	1 (2) ^b^	0 ^b^	0.01	0.01

^1^ Study group enrollment criteria used: Low iron stores if inflammation-unadjusted serum ferritin ≤ 40 µg/L; and iron sufficient non-anemic if inflammation-unadjusted serum ferritin > 40 µg/L and hemoglobin concentration ≥115 g/L. ^2^ Associations of the factors HIV and iron status with the hematological indices were assessed using two-way analysis of variance (ANCOVA) for continuous variables and two-way logistic regression analysis for categorical variables, adjusting for age and sex. Between-group differences were analyzed using one-way ANOVA or Chi-square tests with Bonferroni adjustment for multiple comparisons. Means in a row without a common letter (a, b, c) differ significantly, *p* < 0.05. ^3^ Mean (standard deviation) (all such values). ^4^ Total anemia: hemoglobin < 115 g/L for children 8 to 11 years old, or <120 g/L for children 12 to 13 years old. Moderate anemia: hemoglobin 80 to 109 g/L. Mild anemia: hemoglobin 110 to 114 g/L for children 8 to 11 years old, and 110 to 119 g/L for children 12 to 13 years old [26]. ^5^ Inflammation-adjusted plasma ferritin < 15 µg/L and/or sTfR > 8.3 mg/L, and anemia. ^6^ Significant HIV × iron status interaction effect for mean corpuscular volume (MCV): *p* = 0.01. ^7^ In 8 to 12-year-olds: Microcytosis: MCV < 77.1 fL. Normocytosis: MCV 77.1–91.5. Macrocytosis: MCV > 91.5 fL. In 13-year-old males: Microcytosis: <83.1 fL. Normocytosis: MCV 83.1–101.6 fL. Macrocytosis: MCV > 101.6. In 13-year-old females: Microcytosis: MCV < 78.9 fL. Normocytosis: MCV 78.9–98.5 fL. Macrocytosis: >98.5 fL (cut-off values used by the National Health Laboratory Service in South Africa). ^8^ Microcytic anemia: Microcytosis and anemia. Normocytic anemia: Normocytosis and anemia. Macrocytic anemia: Macrocytosis and anemia.

**Table 5:** Daily nutrient intake in the four groups of South African children purposefully selected according to HIV and iron status.

	Estimated Average Requirements (EAR) ^1^	HIV+ and Low Iron Stores ^2^	HIV+ and Iron Sufficient Non-Anemic	HIV-ve and Low Iron Stores	HIV-ve and Iron Sufficient Non-Anemic	*p*-Value ^3^		
		*n* = 43	*n* = 41	*n* = 45	*n* = 45	HIV	Iron Status	HIV × Iron Status
Total protein,^4^ g		69 (53–76) ^ab^	62 (52–76) ^b^	80 (63–102) ^ab^	83 (66–103) ^a^	0.001	0.86	0.38
Total protein <EAR, *n* (%)	0.76 g/kg	0	1 (2)	0	0	-	-	-
Plant protein, g		34 (29–45)	37 (25–42)	37 (30–44)	34 (23–41)	0.66	0.048	0.59
Animal protein, g		32 (22–39) ^bc^	27 (21–37) ^c^	41 (27–54) ^ab^	46 (35–64) ^a^	<0.001	0.35	0.09
Muscle protein, g		21 (15–27) ^b^	20 (15–27) ^b^	28 (18–37) ^ab^	31 (24–49) ^a^	<0.001	0.45	0.26
Iron, mg		16 (14–21)	17 (14–21)	18 (15–21)	17 (13–21)	0.69	0.22	0.94
Iron < EAR, *n* (%)	Boys: 4.1/5.9 mgGirls: 4.1/5.7 mg	0	0	0	0	-	-	-
Heme iron, mg		2.3 (1.6–4.0) ^b^	2.0 (1.5–3.4) ^b^	3.3 (2.0–4.4) ^ab^	3.3 (2.5–5.2) ^a^	<0.001	1.00	0.13
Non-heme iron, mg		13 (12–18)	14 (11–17)	15 (12–17)	13 (10–17)	0.49	0.15	0.70
Iron from fortified foods, mg		8.8 (7.0–12.6) ^a^	10.2 (6.8–12.8) ^a^	8.6 (5.2–12.1) ^a^	6.1 (4.1–8.6) ^b^	0.002	0.03	0.02
Zinc, mg		13 (11–15)	13 (10–16)	14 (11–18)	13 (11–16)	0.25	0.37	0.71
Zinc from fortified foods, mg		6.9 (4.7–8.9) ^a^	7.2 (4.4–10.3) ^a^	5.7 (4.1–8.7) ^a^	3.7 (2.5–5.9) ^b^	<0.001	0.06	0.007
Zinc < EAR, *n* (%)	4.0/7.0 mg	0	1 (2)	1 (2)	2 (4)	-	-	-
Calcium, mg		454 (311–582) ^bc^	399 (312–536) ^c^	584 (410–775) ^ab^	664 (522–951) ^a^	<0.001	0.35	0.09
Calcium < EAR, *n* (%)	800/1100 mg	42 (98)	40 (98)	43 (96)	39 (87)	0.13	0.21	0.54
Vitamin A, µg RAE ^5^		409 (239–820) ^b^	536 (284–1017) ^ab^	582 (354–990) ^ab^	809 (408–1486) ^a^	0.06	0.02	0.70
Vitamin A < EAR, *n* (%)	Boys: 275/445 µg RAEGirls: 275/420 µg RAE	23 (54)	15 (37)	14 (31)	12 (27)	0.049	0.11	0.34
Thiamin, mg		1.7 (1.4–2.1)	1.8 (1.4–2.1)	2.1 (1.5–2.5)	1.8 (1.3–2.4)	0.25	0.27	0.37
Thiamin < EAR, *n* (%)	0.5/0.7 mg	0	0	0	0	-	-	-
Riboflavin, mg		1.3 (1.0–1.8) ^b^	1.2 (0.9–1.6) ^b^	1.6 (1.2–1.9) ^ab^	1.9 (1.2–2.4) ^a^	<0.001	0.25	0.08
Riboflavin < EAR, *n* (%)	0.5/0.8 mg	4 (9)	3 (7)	4 (9)	4 (9)	0.78	0.73	0.69
Niacin, mg		26 (23–33)	26 (21–35)	31 (25–36)	29 (22–34)	0.18	0.31	0.92
Niacin < EAR, *n* (%)	6/9 mg	0	0	0	0	-	-	-
Vitamin B_6_, mg		4.6 (3.8–6.1)	5.0 (3.4–6.1)	4.7 (3.7–5.7)	4.0 (3.0–5.6)	0.21	0.13	0.51
Vitamin B_6_ < EAR, *n* (%)	0.5/0.8 mg	0	0	0	0	-	-	-
Folate, µg		426 (362–553)	453 (335–609)	458 (355–543)	420 (295–531)	0.39	0.33	0.46
Folate < EAR, *n* (%)	160/250 µg	1 (2)	2 (5)	4 (9)	6 (13)	0.06	0.39	0.88
Vitamin B_12_, µg		5.7 (3.5–9.0) ^b^	4.5 (3.5–7.7) ^b^	8.6 (4.7–12.4) ^ab^	9.7 (5.8–13.9) ^a^	<0.001	0.72	0.31
Vitamin B_12_ < EAR, *n* (%)	1/1.5 µg	0	0	0	1 (2)	-	-	-
Vitamin C, mg		59 (38–86) ^bc^	36 (28–52) ^c^	74 (29–158) ^ab^	85 (59–163) ^a^	<0.001	0.96	0.004
Vitamin C < EAR, *n* (%)	22/39 mg	11 (26) ^bc^	24 (59) ^a^	17 (38) ^ab^	4 (9) ^c^	0.20	0.003	<0.001
Total fiber, g		25 (20–31)	25 (19–30)	28 (22–37)	25 (18–35)	0.40	0.09	0.85
Total fiber < AI,^6^ *n* (%)	Boys: 25/31 gGirls: 25/26 g	26 (61) ^ab^	31 (76) ^a^	18 (40) ^b^	27 (60) ^ab^	0.04	0.03	0.54

^1^ The estimated average requirements (EAR) values established for 4 to 8-year-old/9 to 13-year-old age categories [40,41]. ^2^ Study group enrollment criteria used: Low iron stores if inflammation-unadjusted serum ferritin ≤ 40 µg/L; and iron sufficient non-anemic if inflammation-unadjusted serum ferritin > 40 µg/L and hemoglobin concentration ≥ 115 g/L. ^3^ Associations of the factors HIV and iron status with daily nutrient intakes were assessed using two-way analysis of variance (ANCOVA) for continuous variables and two-way logistic regression analysis for categorical variables, adjusting for age and sex. Between-group differences were analyzed using one-way ANOVA or Chi-square tests with Bonferroni adjustment for multiple comparisons. Non-normally distributed variables were log-transformed prior to analysis. Medians in a row without a common letter (a, b, c) differ significantly, *p* < 0.05. ^4^ Median (inter-quartile range) (all such values). ^5^ RAE, retinol activity equivalents (1 RAE = 1 µg retinol = 12 µg beta-carotene = 24 µg α-carotene) [40]. ^6^ No EAR established; adequate intake (AI) values used [40].

## Data Availability

The data presented in this study are available on request from the corresponding author.

## Appendix A

**Table A1:** Antiretroviral regimens (*n* = 84).

Regimen		HIV+ and Low Iron Stores ^1^	HIV+ and Iron Sufficient Non-Anemic	Total
		*n* = 43	*n* = 41	*n* = 84
ABC-3TC-LPV/r		23 (53)	18 (44)	41 (49)
ABC-3TC-EFV		14 (33)	5 (12)	19 (23)
AZT-3TC-LPV/r		5 (12)	11 (27)	16 (19)
AZT-3TC-NVP		0	3 (7)	3 (4)
ABC-3TC-ATV/r	*n* (%)	0	1 (2)	1 (1)
AZT-3TC-EFV		0	1 (2)	1 (1)
3TC-EFV-LPV/r		0	1 (2)	1 (1)
TDF-FTC-EFV		0	1 (2)	1 (1)
TDF-FTC-LPV/r		1 (2)	0	1 (1)

^1^ Study group enrollment criteria used: Low iron stores if inflammation-unadjusted serum ferritin ≤ 40 µg/L; and iron sufficient non-anemic if inflammation-unadjusted serum ferritin > 40 µg/L and hemoglobin concentration ≥115 g/L. Antiretroviral regimens: Nucleoside reverse transcriptase inhibitors (NRTIs) include: abacavir (ABC), lamivudine (3TC), zidovudine (AZT), tenofovir (TDF) and emtricitabine (FTC). Non-nucleoside reverse transcriptase inhibitors (NNRTIs) include: efavirenz (EFV) and nevirapine (NVP). Protease inhibitors (PI) include: lopinavir boosted with ritonavir (LPV/r) and atazanavir boosted with ritonavir (ATV/r).

## Appendix B

**Table A2:** Most eaten food items from the abbreviated quantified food frequency questionnaire by all children (*n* = 174).

Rank	Ranked by Proportion of Children			Ranked by Mean Daily Intake		
	Item	Proportion of Children Who Reported Consumption (%)	Mean Daily Intake Per Person (g)	Item	Proportion of Children Who Reported Consumption (%)	Mean Daily Intake per Person (g)
1	Bread, white and brown	100	180	Maize meal porridge, cooked	94	232
	Potato	100	88			
2	Chicken	99	20	Bread, white and brown	100	180
3	Milk	98	159	Milk	98	159
4	Apple	96	62	Orange, peeled	59	96
5	Maize meal porridge, cooked	94	232	Potato	100	88
6	Egg	90	20	Soup (vegetable or bean)	67	72
	Baked beans	90	18			
7	Tinned fish	87	19	Mageu ^2^	23	64
8	Carrots	86	7	Apple	96	62
9	Peanut butter	84	17	Tangerine, peeled	39	61
	Polony ^1^	84	16			
	Fresh or frozen fish	84	8			
10	Cabbage	82	9	Oats, cooked	32	47

^1^ Cold meat sausage made from a combination of beef and pork. ^2^ Drinkable fermented maize porridge.
